# Production of acetoin and its derivative tetramethylpyrazine from okara hydrolysate with *Bacillus subtilis*

**DOI:** 10.1186/s13568-023-01532-z

**Published:** 2023-02-28

**Authors:** Tao Li, Ping Liu, Gege Guo, Zhaoxing Liu, Lei Zhong, Lianxia Guo, Cheng Chen, Ning Hao, Pingkai Ouyang

**Affiliations:** State Key Laboratory of Materials-Oriented Chemical Engineering, Jiangsu National Synergetic Innovation Center for Advanced Materials (SICAM), College of Biotechnology and Pharmaceutical Engineering, Nanjing Tech University, Nanjing, 211816 China

**Keywords:** Okara, Acetoin, Tetramethylpyrazine (TTMP), *Bacillus subtilis*, Galactose

## Abstract

Okara, a renewable biomass resource, is a promising fermentative raw material for the bio-production of value-added chemicals due to its abundance and low-costs. we developed a process for the enzymatic hydrolysis of okara, and then engineered *Bacillus subtilis* to utilize mixed sugars to produce acetoin in okara hydrolysis without the addition of a supplemental nitrogen source. Okara was initially hydrolyzed with cellulase, β-glucosidase, and pectinase to obtain okara hydrolysate containing mixed sugars (32.78 ± 0.23 g/L glucose, 1.43 ± 0.064 g/L arabinose, 7.74 ± 0.11 g/L galactose) and amino acids. In this study, *Bacillus subtilis* 168 was used as the acetoin-producing strain, and the key genes *bdhA* and *acoA* of the acetoin catabolism pathway were knocked out to improve the fermentation yield of acetoin. In order to utilize the galactose in the hydrolysate, the recombinant strain BS03 (*Bacillus subtilis*168∆*bdhA*∆*acoA*) was used to overexpress the arabinose transporter-encoding gene (*araE*) drive heterologous expression of the Leloir pathway gene (*galKTE*). The corn dry powder concentration was optimized to 29 g/L in the reducing sugar okara hydrolysate. The results show that the recombinant bacterium BS03 could still synthesize 11.79 g/L acetoin without using corn dry powder as a nitrogen source. Finally, using okara enzymatic hydrolysate as the carbon and nitrogen source, 11.11 g/L and 29.7 g/L acetoin were obtained by batch fermentation and fed-batch fermentation, respectively, which was further converted to 5.33 g/L and 13.37 g/L tetramethylpyrazine (TTMP) by reaction with an ammonium salt.

**Supplementary Information:**

The online version contains supplementary material available at 10.1186/s13568-023-01532-z.

## Introduction

Lignocellulosic is renewable biomass resource used in fermentation to produce biofuels and platform chemicals feedstocks (Martín et al. 2002). Among potential lignocellulosic biomasses, okara is a promising, readily available, low-cost renewable resource (Cui et al. 2021). As a by-product of the processing of soymilk and tofu, okara has a wide range of sources (2.8 million tons of okara waste are produced annually in China alone) and is rich in nutrients (100 g dried okara contains 42.4–58.1% carbohydrate, 15.2–33.4% protein and 8.3–10.9% crude fat) (Redondo-Cuenca et al. 2008; Su et al. 2021). In previous studies, okara was generally used for solid-state fermentation of fungi and bacteria to extract bioactive substances or to produce directly edible foods (Vong and Liu 2016). Few studies have focused on preparing fermentable sugar from okara to produce microbial fermentation chemicals. Choi et al. (2015) used various fungal species to produce cellulase, hemicellulose, and pectinase to hydrolysis okara as valorization biomass for bioethanol production. Xu et al. (2022) use commercial cellulase and hemicellulose hydrolysis okara to produce lactic acid. However, these studies were limited to the utilization of sugar and did not consider the simultaneous utilization of nitrogen components in okara. Considering that okara also contains 15.2–33.4% crude protein, the utilization of okara as a nitrogen source has needs to be further investigated.

Acetoin (3-hydroxy-2-butanone) is an important four-carbon platform compound that is used to synthesize pharmaceuticals, flavorants, and multifunctional materials (Jia et al. 2018). *Bacillus subtilis* is a natural producer of acetoin, and can utilize many crude materials due to the variety of carbohydrate metabolism genes it possesses (Xiao and Lu 2014). Therefore, *Bacillus subtilis* is also an ideal chassis for acetoin production. For example, Zhang et al. (2016) overexpressed the *168* endogenous arabinose transporter (*araE*) and exogenous xylose isomerase genes (*xylA* and *xylB*) in *B. subtilis*; the strain was able to use glucose, xylose, and arabinose simultaneously from lignocellulose for the fermentative production of acetoin. Jia et al. (2017) used heat-tolerant *Bacillus subtilis* IPE5-4 as the acetoin-producing strain to produce 22.76 g/L acetoin from alkali pre-treated corncobs in a 5-L fermenter via the simultaneous saccharification and fermentation (SSF) process. Although a significant amount of research and practical application of okara biomass has been demonstrated, there exist very few studies directly addressing the potential of okara as a cheap carbon and nitrogen source for the conversion of bioproducts.

In this study, we optimized the multi-enzyme hydrolysis of okara raw materials and obtained okara hydrolysate rich in mixed sugars (including glucose, galactose, arabinose) and amino acids. In order to fully convert the carbon sources in okara hydrolysate into acetoin through microbial fermentation, we constructed a recombinant strain BS03 strain that effectively utilized arabinose and galactose obtained from the okara hydrolysate. Through further optimization of the concentration of hydrolysate, we were able to obtain a high acetoin yield with supplementing corn dry powder as a nitrogen source. These results demonstrate that okara enzymatic hydrolysate is a good carbon source and an excellent nitrogen source for acetoin fermentation. Finally, in a 7.5L bioreactor, we established a fermentation process route for acetoin production from okara hydrolysate. This study provides a reference for the commercial production of acetoin from renewable biomass resources.

## Material and methods

### Experimental material

Okara samples were obtained from Nanjing Guoguo Food Co. Ltd. (Nanjing, China). The wet okara was dried at 80 °C to a constant weight and then pulverised using a pulveriser (HangZhou XuZhong Food Machinery Co., Ltd, Hangzhou, China) and filtered through a 40-mesh sieve for later use. Cellulase (130 FPU/g) (Cellic CTec2), β-glucosidase (45 FBG/g) (UltraFLO L), xylanase (500 FXU-S/g) (Shearzyme 500 L), and pectinase (3300 PGNU/g) (Pectinex UF) were purchased from Novozymes China (Beijing) Investment Company. Para-chloro-phenylalanine (*p*-Cl-Phe; Lot No. SHBC0245V) was purchased from Sigma-Aldrich (Nanjing, China). All analytical reagents were purchased from Nanjing Wanqing Chemical Glass Wear & Instrument Co. Ltd.

### Strains and plasmids

The strains and plasmids used in this study are listed in Tables 1. *E. coli* DH5α and MG1655 were used for plasmid amplification and the source (preparation) of the galactose metabolism gene cluster *galKTE*, respectively. The host strain *B. subtilis* 168 and pheS* counter-screening markers were provided by Prof. Yan Xin of Nanjing Agricultural University (Nanjing, China). The *E. coli*-*B. subtilis* shuttle plasmid pMA5 was purchased from Wuhan Miaoling Biotechnology Co., Ltd. (Miaoling, Wuhan, China).

**Table 1:** Strain and plasmid used in study

Name	Characteristic	Ref.
Strain		
JM109	recA1, endA1, gyrA96, thi, hsdR17, supE44, relA1, Δ (lac proAB)/F, [traD36, proAB + , lacqlacZ∆M15]	Takara
BS168	*Bacillus subtilis*168	Lab stock
BS01	*Bacillus subtilis*168 derivate, *BS*168∆*bdhA*, ∆*acoA*	This study
BS02	BS01 derivate, expressing *araE* gene under the control of *HpaII* promoter via the plasmid pMA5	This study
BS03	BS01 derivate, expressing *araE*,*galKTE* gene under the control of the *HpaII* promoter via the plasmid pMA5	This study
BS04	BS01 derivate, BS01 ∆*ptsG*, ∆*yyzE*, ∆*ypqE*, expressing *araE*, *galKTE* gene under the control of *HpaII* promoter via the plasmid pMA5	This study
Plasmids		
pMA5	Amp, Kana, *E.coli*-*Bacillus* shuttle vector, *HpaII* promoter	Lab stock
pMA5-*araE*	pMA5 harboring *araE* gene under the control of *HpaII* promoter	This study
pMA5-*araE*-*galKTE*	pMA5 harboring *araE, galKTE* gene under the control of *HpaII* promoter	This study

### Plasmid construction and transformation

The primers used in this study are listed in Additional file 1: Table S1. The *B. subtilis* 168 and *E. coli* MG1655 genomes were extracted using the TIANamp Bacteria DNA Kit (Tiangen, Beijing, China). The arabinose transporter-encoding gene *araE* and galactose-related gene cluster *galKTE* (Gene ID:945358,945357,945354) were amplified from the genomes of *B. subtilis* 168 and *E. coli* MG1655, respectively, using 2 × Phanta Max Master Mix DNA polymerase (Vazyme, Nanjing, China). The pMA5 plasmid was digested with restriction enzymes *Bam*HI and *MIu*I (Takara, Beijing, China), and each gene was ligated into the vector using the ClonExpress Ultra One Step Cloning Kit (Vazyme, Nanjing, China). The recombinant plasmid was transformed into *B. subtilis* using the Spizizen method (Vojcic et al. 2012).

**Fig. 1:**
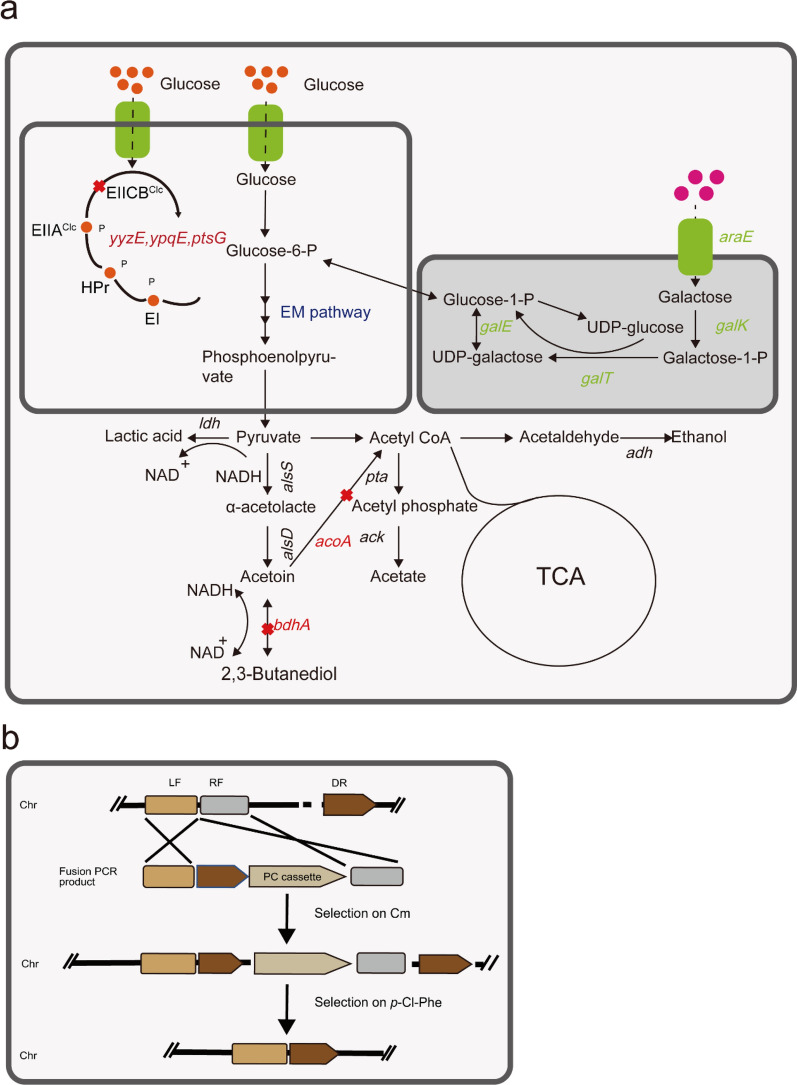
**a** Overview of the production of TTMP from a mixture of glucose and galactose by *B. subtilis* engineered bacteria. Green words represent genes expressed on plasmid pMA5. Red words represent knockout genes. **b** pheS* counter-screening marker gene knockout process

The knockdown procedure was based on previous work by Zhou et al. (2017) (Fig. 1b). Primers were used to amplify the upstream (~ 800 bp) (LF) and the downstream homologous arm (~ 800 bp) (RF) of the deleted fragment, repeat sequence DR (~ 500 bp), and PC cassette (*P*_*bc*_-pheS* -*cat* cassette) (~ 1900 bp). LF, DR, PC, and RF were fused using overlapping PCR. Positive clones were screened on 5 μg/mL erythromycin plates, and subsequently cultured in a non-resistant LB medium until the OD_600_ reached 1 and coated with sterile water diluted 100-fold onto MGY-Cl (5 g/L glucose, 4 g/L yeast extract, 1 g/L NH_4_NO_3_, 0.5 g/L NaCl, 1.5 g/L K_2_HPO_4_, 0.5 g/L KH_2_PO_4_, 0.2 g/L MgSO_4_, 5 mM *p*-Cl-Phe, and 20 g/L agar powder) on solid medium. The knockout strains were verified using PCR and sequencing.

### Preparation of okara hydrolysate

#### Enzymatic saccharification of acid-pretreated okara

The dried okara was treated with 500 mM sulphuric acid for 1.5 h at 121 °C, with a loading rate of 6 g/50 mL. After pretreatment, the pH was adjusted to 4.8 using 3M NaOH, and enzymatic hydrolysis was carried out for 36 h with cellulase loading at 8 FPU/mL, the supernatant was collected after centrifugation for 15 min at room temperature, 6000 rpm, and used for analysis.

#### Enzymatic hydrolysis of okara

The enzymatic hydrolysis was performed in a 250-mL conical flask with a working volume of 50 mL and a solid-to-liquid ratio of 6 g/50 mL. The pH of the enzymatic hydrolysis system (cellulose, β-glucosidase, xylanase, and pectinase) was controlled between 4.8 and 5.0 using a citrate-sodium citrate buffer (50 mM). The conical flask was placed in a water bath shaker (Henan Bainian Instrument Co., Ltd.) at 50 °C and 150 rpm for 36 h during the reaction. At the end of hydrolysis, the samples were autoclaved at 105 °C for 10 min to inactivate the enzymes. The supernatant was centrifuged (room temperature, 6000 rpm, 15 min) and used for analysis and fermentation. A rotary evaporator was used to concentrate the enzymatic hydrolysate for the bioreactor fermentation scale-up.

#### Medium and culture conditions

Seed liquid was cultured in LB medium at a volume of 100/500 mL. Fermentation experiments were performed in a 50/250-mL conical flask at 37 °C using M9 medium and acetoin production meduium (carbon source, 15 g/L corn dry power, 3 g/L urea, pH 6.8 after sterilization) for strain fermentation with a rotating speed of 100 rpm.

#### Batch fermentation of individual sugars

Batch fermentations were carried out in a conical flask with 10 g/L of commercial monosaccharides (glucose, xylose, galactose, and arabinose) as the carbon source, 15 g/L corn dry powder, and 3 g/L urea.

#### Acetoin production using conical flask fermentation

The effect of an initial reducing sugar concentration in the hydrolysate (with a range of 8 g/L to 41 g/L and supplemented with 1.5% corn dry powder and 0.3% urea) on acetoin production was investigated by conical flask fermentation. Subsequently, optimization of corn dry powder with a concentration range of 0–3% and 29 g/L reducing sugar containing hydrolysate as the carbon source was carried out for acetoin production.

#### Bioreactor culture

After optimization of the shake flask culture (29 g/L reducing sugar containing hydrolysate, 0.3% urea), batch and fed-batch fermentations were performed in a 7.5-L fermenter with a working volume of 3 L. The pH, temperature, and rotation speed were 6.8, 37 °C, and 400 rpm, respectively. The inoculum volume was 5% (v/v), and the aeration ratio was 1 vvm (air volume/culture volume/min).

#### TTMP synthesis

(NH_4_)_2_HPO_4_ and acetoin were mixed in a molar ratio of 3:1 and reacted at 105 °C for 3 h to obtain TTMP as previously described (Peng et al. 2020).

#### Scanning electron microscopy (SEM)

A Hitachi S-4800 (Japan) scanning electron microscope (SEM) was used to photograph the surface morphological changes of okara before and after enzymatic hydrolysis.

#### Analysis method

The fundamental components of okara, cellulose, hemicellulose, and lignin were determined using the Van Soest method (Syaftika and Matsumura 2018). The crude protein content was determined using the Kjeldahl method as per the Chinese national standard GB 5009.5–2016. Pectin and ash were measured as per the Chinese national standard GB 25533-2010 and GB/T 5505-2008, respectively. Organic acids (succinic, acetic, and lactic acids), acetoin, and 2,3-butanediol were detected by liquid chromatography on an Agilent 1290 Infinity (Agilent Technologies, Waldbronn, Germany) instrument. The column model was an Aminex HPX-87H (Bio-Rad), injection volume was 20 μL, mobile phase was 5 mM H_2_SO_4_, flow rate was 0.6 mL/min, column temperature was 60 °C, and a refractive index detector was used (Souza et al. 2021). TTMP was determined as per Peng et al. (2020). The mobile phase included 0.1% formic acid and acetonitrile mixed at a ratio of 8:2 (v/v), the flow rate was 0.8 mL/min, injection volume was 10 μL, column temperature was 40 °C, and column used was an XBridge C18 (5 μm, 4.6 × 250 mm). The detector wavelength was 278 nm. Monosaccharides were determined using a Shodex SUGAR SP-G 6B (6.0 mm I.D. × 50 mm) column; the mobile phase was double distilled water, flow rate was 0.5 mL/min, column temperature was 80 °C, injection volume was 10 μL, and a refractive index detector was used (Ding et al. 2019). Amino acid content was determined using PITC pre-column derivatisation (Hao et al. 2016; Li et al. 2021), and the conditions were as follows: column model Hedera ODS-2 column (4.6 mm × 250 mm, 5 μm, Hanbon Sci. & Tech., Jiangsu, China); UV detector wavelength 254 nm; column temperature 40 °C, flow rate 1 mL/min, injection volume 10 μL; mobile phase A, 0.1 mol/L sodium acetate pH 6.5; mobile phase B, 80% acetonitrile; gradient elution, (5 min 3%B, 14 min 11%B, 17 min 21% B, 29 min 34% B, 41 min 100% B, 43 min 100% A, 47 min 100% A). Biomass was determined by a spectrophotometer (756S, China) at 600 nm. The cell dry weight using the empirical formula 1 OD_600_ = 0.352 DCW (g/L) (Hu et al. 2020).

## Results

### Optimisation of the enzymatic hydrolysis of okara

**Fig. 2:**
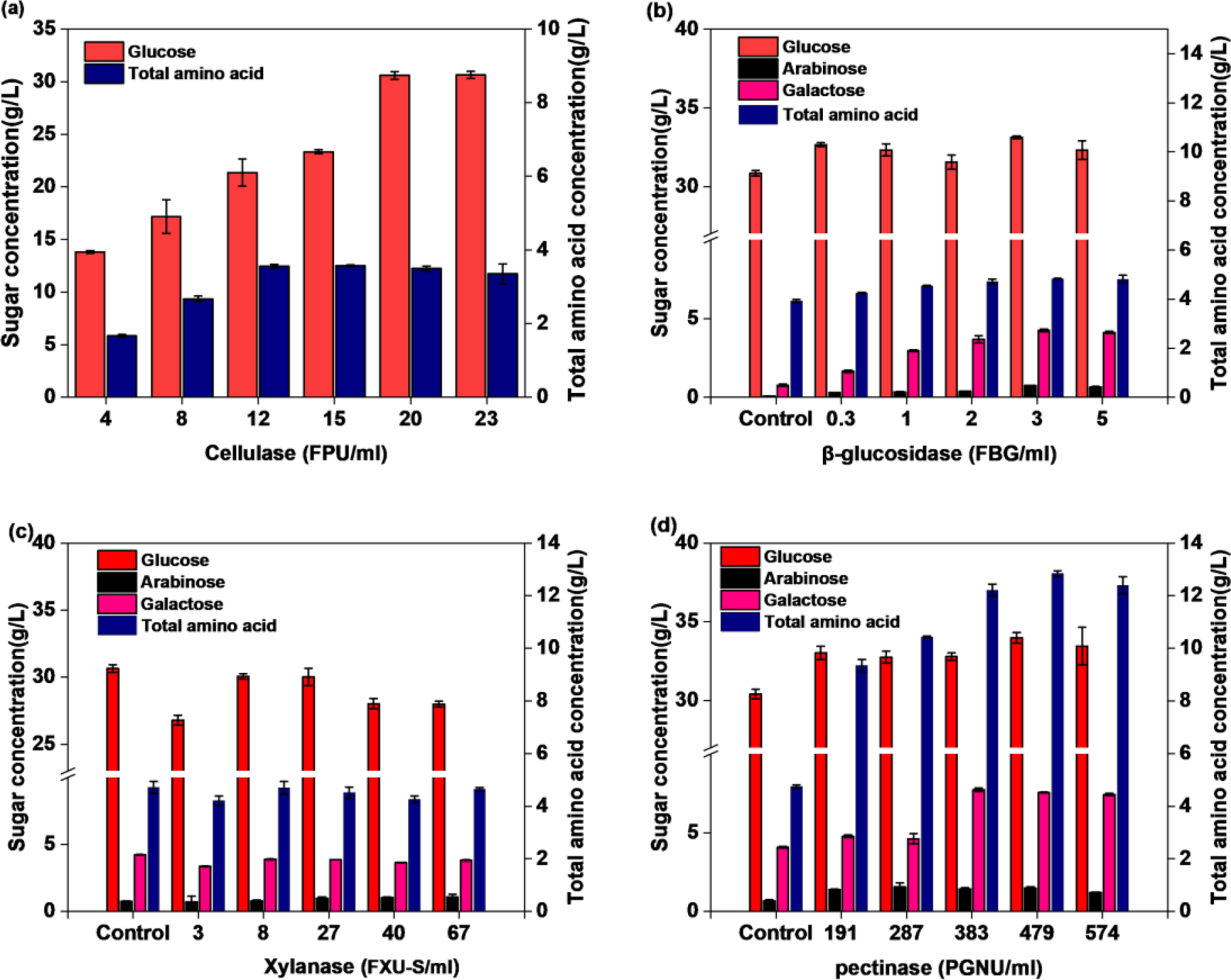
Effects of addition of cellulase, β-glucosidase, xylanase, and pectinase on the dissolution of monosaccharides and amino acids from okara. **a** Cellulase; **b** 20 FPU/mL cellulase and β-glucosidase; **c** 20 FPU/mL cellulase, 3 FBG/mL β-glucosidase and xylanase; **d** 20 FPU/mL cellulase, 3 FBG/mL β-glucosidase and pectinase

The composition of dry okara is shown in Table 2. The main components of okara include 28.3% cellulose, 17.15% hemicellulose, 12.3% lignin, 2.36% pectin, 17.5% crude protein and 3% ash. The remaining undetected components have been previously described as mainly some crude fat, malonyl glucoside, isoflavone glucoside, isoflavone glycoside, etc. (Vong and Liu 2016). Two methods were used to treat okra to prepare the hydrolysate: the first method, the okara acid hydrolysate was obtained after pre-treated with high-temperature dilute sulphuric acid, followed by cellulase hydrolysis. The carbohydrate concentrations (25.25 g/L glucose, 14.6 g/L galactose, 7.56 g/L arabinose, 4.1 g/Lxylose, 2.1 g/L mannose) of the okara acid hydrolysate are shown in Table 2. Dilute acid hydrolysis is a fast and specific method to break-down the hemicellulosic fraction of cell wall into their monomeric constituents (Moutta et al. 2012). During the acid catalyzed process at high temperature, hemicelluloses fraction of cell wall is depolymerised into simple sugars. The second method, without pre-treatment, reduce sugars (32.78 ± 0.23 g/L glucose, 1.43 ± 0.064 g/L arabinose, 7.74 ± 0.11 g/L galactose) and amino acids in okara can also be obtained by enzymatic hydrolysis (cellulase, β-glucosidase, xylanase, and pectinase) (Fig. 2d).

**Table 2:** Dried okara and okara acid hydrolysate components

Component	Dry okara (%)
Cellulose	28.3 ± 0.14
Hemicellulose	17.15 ± 0.35
lignin	12.3 ± 0.28
Pectin (Calculated as galacturonic acid)	2.365 ± 0.17
Crude protein	17.5 ± 0.56
Ash	3 ± 0.14

Acid + Cellulase	Okara hydrolysate (g/L)
Glucose	25.25 ± 0.63
Galactose	14.6 ± 0.42
Xylose	4.1 ± 0.09
Arabinose	7.56 ± 0.14
Mannose	2.10 ± 0.13

**Fig. 3:**
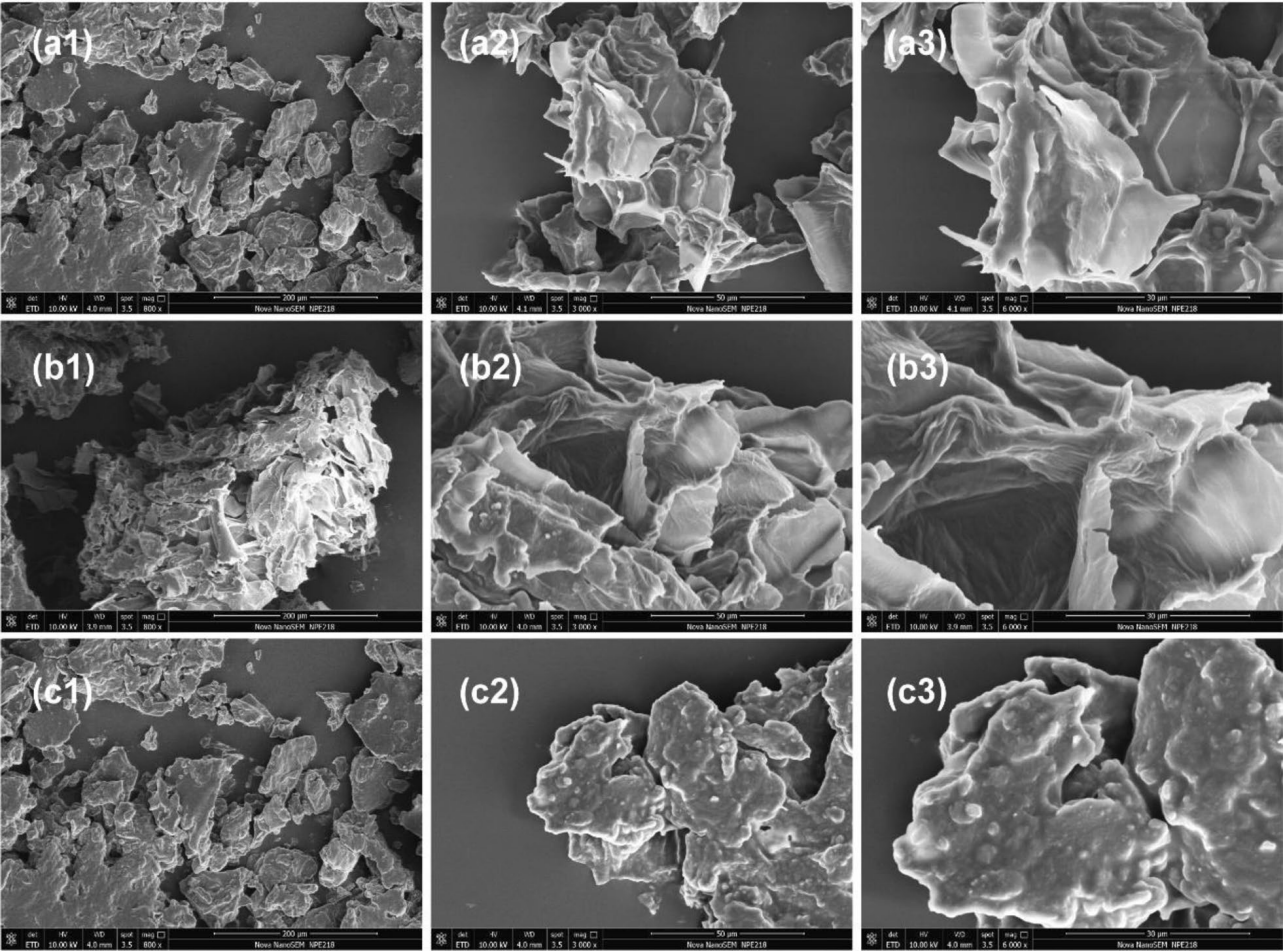
SEM images of okara samples, either untreated dry okara (**a1**, **a2**, **a3**) or treated using cellulase (**b1**, **b2**, **b3**) and cellulase + β-glucosidase + pectinase (**c1**, **c2**, **c3**). The magnification and scale bars are provided in each micrograph

Figure 2 shows the optimization of the enzymatic hydrolysis process for dry okara. At a substrate concentration of 120 g/L, enzyme loading of 20 FPU/ml cellulase resulted in the highest total sugar concentration in the okara hydrolysis solution (approximately 30.58 g/L glucose). Further addition of cellulase did not improve the total sugar content (Fig. 2a). It is well known that cellobiose inhibits the action of exo-glucanase. Supplementation with β-glucosidase enabled further hydrolysis of cellobiose released by cellulase. Addition of β-glucosidase (3 FBG/mL enzyme loading) increased the glucose concentration by 4.79%. Meanwhile, low dissolved concentrations of arabinose (0.74 g/L) and galactose (4.26 g/L), increased the hydrolysis of hemicellulose (Fig. 2b). Figure 2c shows that the xylanase concentration in a range of 3–67 FXU-S/mL did not affect the dissolution of total sugars and amino acids, a result which is consistent with Xu et al. (2022). In dry okara, the pectin component affects the release of okara monosaccharides. Consequently, different concentrations of pectinase were added to the enzymatic hydrolysis system. When the pectinase loading reached more than 383 PGNU/mL, the dissolution of monosaccharides, especially galactose and amino acids, was observed. SEM showed that after the addition of pectinase, the surface structure of okara changed from tight and orderly to loose (Fig. 3). In this study, reducing sugar produced from 120 g/L of okara addition was applied in the following fermentation experiments. Approximately 43 g/L of total sugar (32.78 ± 0.23 g/L glucose, 1.43 ± 0.064 g/L arabinose, 7.74 ± 0.11 g/L galactose) and 12.21 g/L of total amino acids were obtained at this substrate concentration (Fig. 2d, Additional file 1: Table S2).

### Acetoin production by engineered *B. subtilis* cultured in glucose-galactose mixtures

**Fig. 4:**
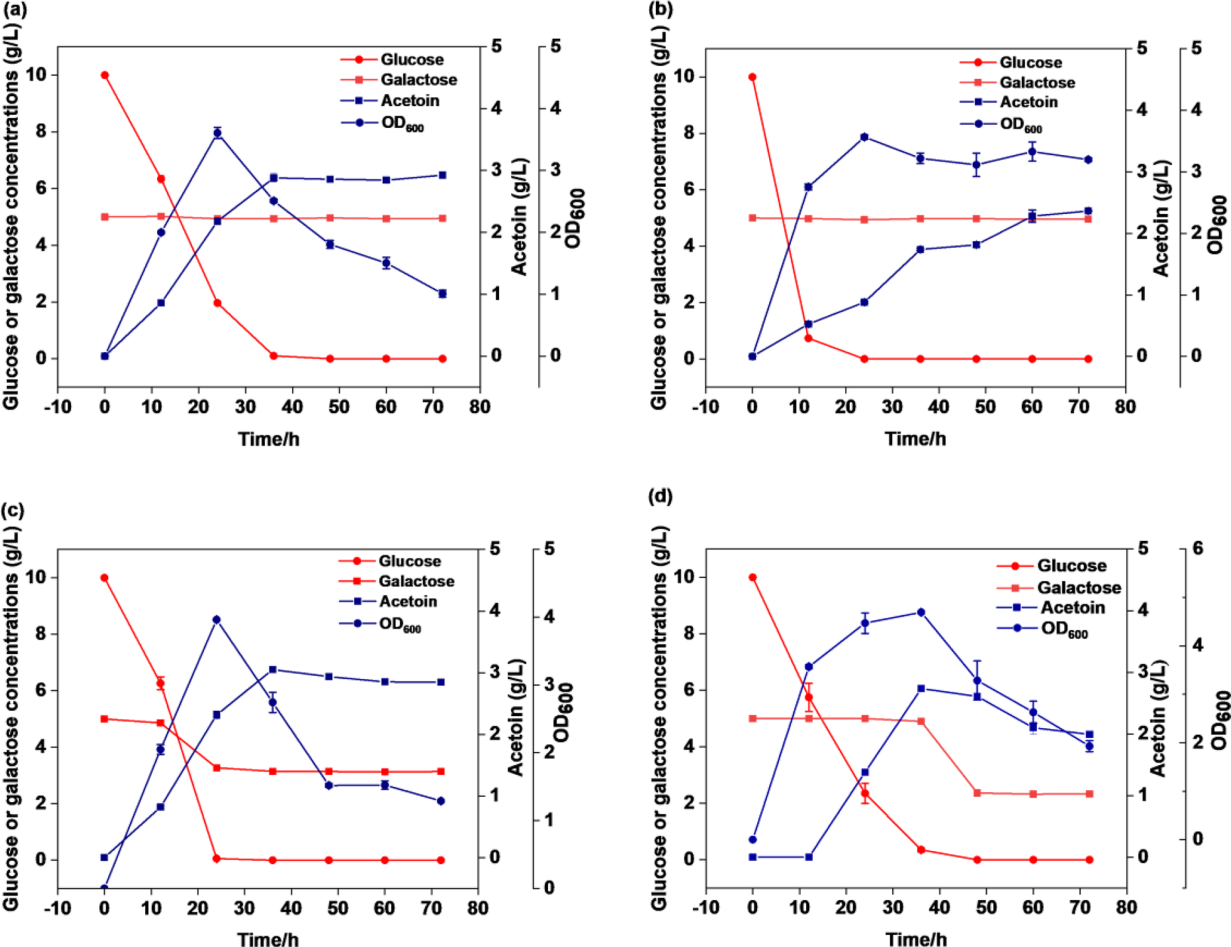
Recombinant strain fermented acetoin under microaerobic conditions in M9 mixed sugar medium (10 g/L glucose + 5 g/L galactose). **a** BS01-pMA5; **b** BS02; **c** BS03; **d** BS04

In this study, the main monosaccharide components of the okara hydrolysate were glucose and galactose (Fig. 2d, Table 2). However, *B. subtilis* 168 cannot grow on galactose and xylose as the sole carbon sources in an M9 minimal salt medium (Chen et al. 2013). The engineered strain BS01 (*B. subtilis* 168∆*bdhA*∆*acoA*) was used as the starting strain for acetoin production (Fig. 1a, Additional file 1: Fig. S2). The engineered strains was fermented in mixed sugar M9 minimal salt medium (10 g/L glucose and 5 g/L galactose), and BS01 containing the empty plasmid pMA5 was used as a control strain. In the co-fermentation of glucose and galactose, BS01-pMA5, BS02, and BS03 completely consumed glucose within 36 h. During this period, the engineered strains BS01-pMA5 and BS02 did not consume galactose (Fig. 4a, b). In the presence of glucose/galactose mixtures diauxic growth was observed with glucose as the preferred substrate. After consuming 1.59 g/L galactose at a rate of 0.13 g/L/h from 12 to 24 h, the biomass decreased sharply. The remaining galactose was not consumed (Fig. 4c), possibly owing to the M9 basic salt medium, which could not meet the bacterial growth requirements of from galactose (Krispin and Allmansberger 1998). We knocked out the key enzymes (*ptsG*, *yyzE*, *ypqE*) in the PTS^Glc^ system of *B. subtilis* (Fig. 1a, Additional file 1: Fig. S1), improving the co-utilization efficiency of galactose and weakening the effect of carbon catabolite inhibition (CCR). The results are shown in Fig. 4d, the glucose consumption rate decreased by 0.414–0.268 g/L/h, the galactose consumption rate increased by 0.13–0.21 g/L/h after inactivating of the PTS system, and the consumption increased by approximately 59.4% to 2.535 g/L (Fig. 4c, d). Although the consumption rate of galactose increased, the fermentation yield of acetoin (3.05 g/L) also decreased by 10.4%. The production rate decreased from 0.0847 to 0.0759 g/L/h.

### Acetoin fermentation using different substrates of the engineered strain under micro-oxygen conditions

**Fig. 5:**
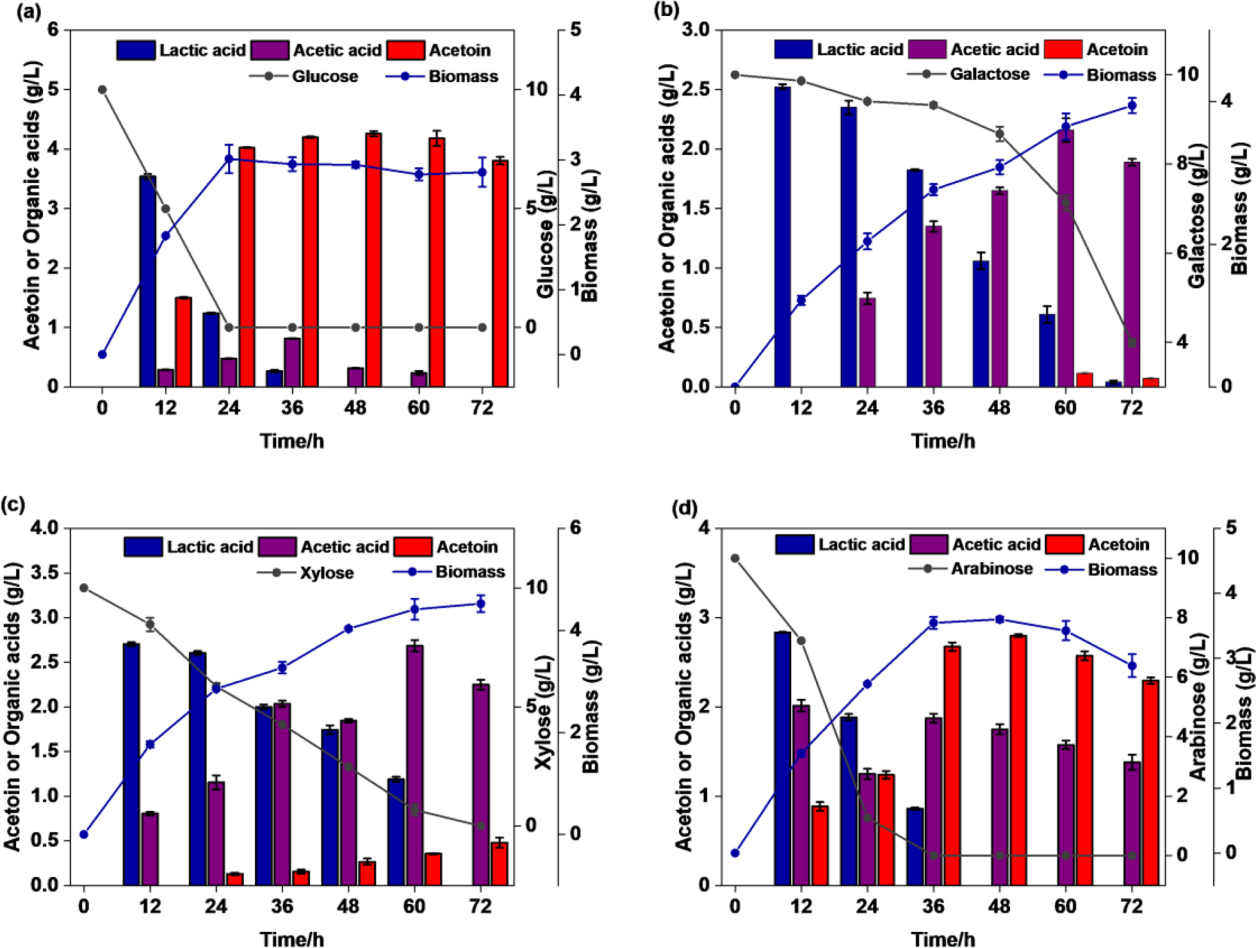
The recombinant BS03 strain was fermented with different monosaccharides to produce acetoin under micro-aerobic conditions. **a** Glucose; **b** Galactose; **c** Xylose; and **d** Arabinose

The monosaccharide components in the okara hydrolysate included xylose and arabinose, in addition to glucose and galactose (Table 2). To evaluate the feasibility of using okara hydrolysate as a cheap carbon source for acetoin fermentation, the monosaccharide components in okara hydrolysate were used as the only carbon source for batch fermentation. As shown in Fig. 5, monosaccharides such as glucose, arabinose, xylose, and galactose were effectively utilized by the recombinant strain BS03. Figures 5a and d show the relationship between acetoin and lactic acid synthesis.

When glucose and arabinose were added as substrates, from 12 to 48 h, the acetoin concentration increased by 2.84-fold and 3.5-fold, respectively, while the lactate concentration gradually decreased from 3.54 g/L and 2.83 g/L to 0 g/L, respectively (Fig. 5a, d). With glucose as the sole carbon source, the concentration and production rate of acetoin were 4.26 g/L and 0.089 g/L/h, respectively, which were slightly higher than those with arabinose (2.8 g/L and 0.058 g/L/h) as the sole carbon source (Fig. 5a, d). When glucose was used as the substrate, the acetic acid concentration dropped to a dropped to an undetectable level (0 g/L) in the later fermentation stage (Fig. 5a). Other carbon sources (xylose, galactose, and arabinose) resulted in higher acetic acid concentrations; galactose and xylose as the substrates resulted in the highest acetic acid concentration of 2.16 g/L and 2.68 g/L in the fermentation broth, respectively. At 72 h, the acetoin concentration using galactose and xylose as substrates was only 0.07 g/L and 0.48 g/L (Fig. 5b, c), and the sugar consumption rate was 0.083 g/L/h and 0.138 g/L/h, respectively.

### Fermentation production of acetoin using okara enzymatic hydrolysate

Compared with okara acid hydrolysate, the proportion of glucose in the okara enzymatic hydrolysate was higher, with no inhibiton (furfural, 5-methylfurfural, and phenolic compounds) caused by the thermal acid pre-treatment (Fig. 2, Table 2). Furthermore, the conversion rate of glucose to acetoin (0.426 g/g) for the engineered strain BS03 was much higher than that of galactose (0.04 g/g; Fig. 5a, b). The low efficiency of galactose to acetoin conversion indicates that using okara enzymatic hydrolysate as a carbon source is more suitable for the biotransformation of acetoin (Table 2, Fig. 2). Table 3 shows acetoin production by the engineered strain BS03 at different concentrations of reducing sugars. The results show that at an initial sugar concentration of 29 g/L under microaerophilic conditions produced approximately 11.05 g/L acetoin, 1.4 g/L 2,3-butanediol, 2.01 g/L acetic acid, and 1.07 g/L succinic acid; the conversion rate of acetoin was the highest (46.08%).

**Table 3:** Acetoin production under varied initial concentrations of reducing sugar

Initial concentrations of reducing sugar (g/L)	Acetoin (g/L)	2,3-Butanediol (g/L)	Acetate (g/L)	Succinic acid (g/L)	Residual reducing sugar concentration (g/L)	Acetoin yield (%)
8	3.12 ± 0.049	ND	ND	ND	0	39
20	8.19 ± 0.037	0.92 ± 0.005	1.27 ± 0.035	0.71 ± 0.025	1.92 ± 0.07	45.29
29	11.05 ± 0.02	1.4 ± 0.015	2.01 ± 0.03	1.07 ± 0.025	5.02 ± 0.06	46.08
41	15.55 ± 0.06	1.89 ± 0.02	2.53 ± 0.04	1.23 ± 0.04	6.85 ± 0.1	45.53

*ND* Not Detected

**Fig. 6:**
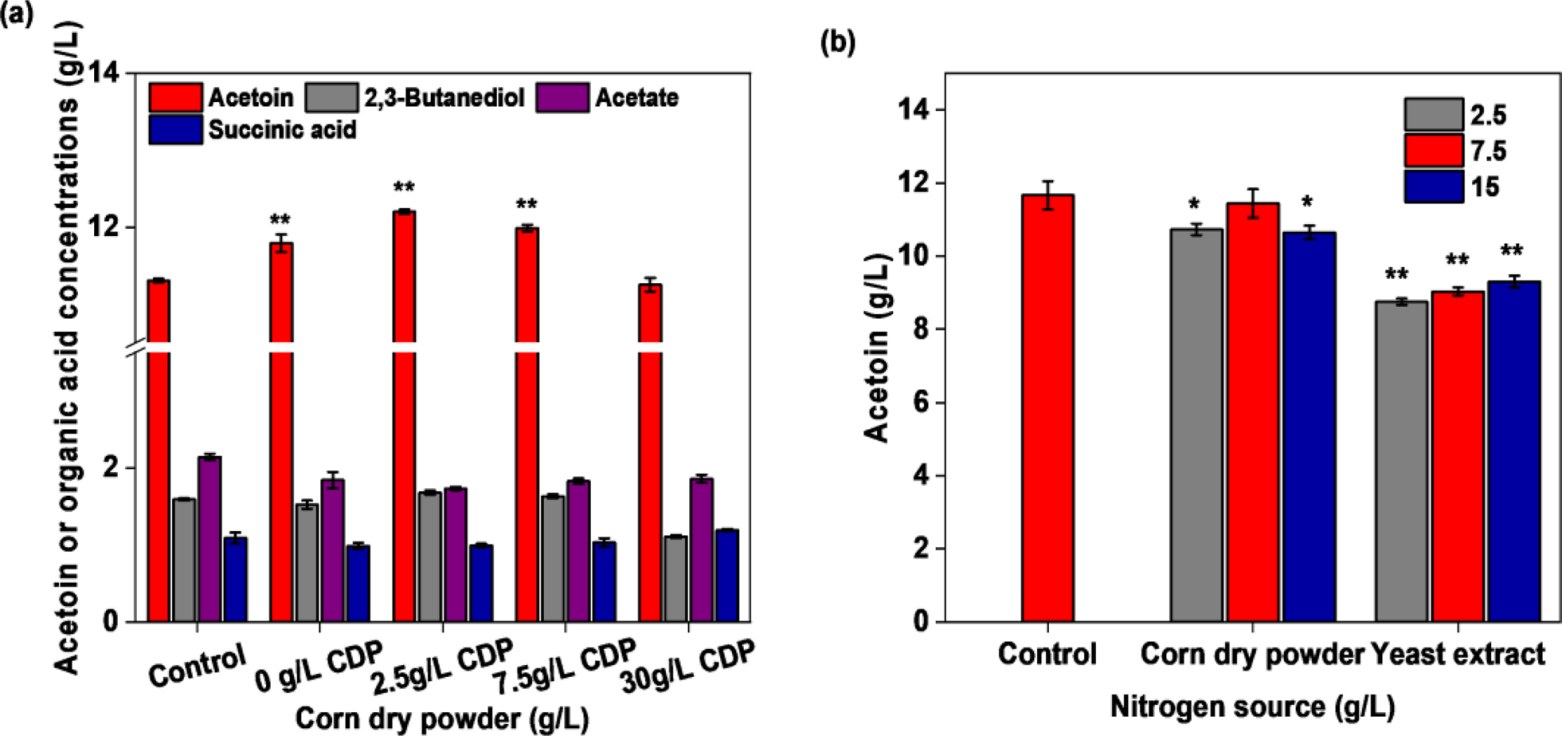
**a** Production of acetoin using okara enzymatic hydrolysate as carbon source in conjunction with different amounts of corn dry powder (CDP). Control: okara enzymatic hydrolysate as the carbon source, 15 g/L corn dry power. **b** Comparison of acetoin fermentation between okara hydrolysate (29 g/L reducing sugar) supplementation of 3 g/L urea and containing 3 g/L urea simulating okara hydrolysate sugar composition (29 g/L reducing sugar), supplemented with (2.5, 7.5, 15 g/L) corn dry powder and (2.5, 7.5, 15 g/L) yeast extract, respectively. Control: okara enzymatic hydrolysate as the carbon source, 3 g/L urea (*Significance code: p < 0.05, **Significance code: p < 0.01)

Corn dry powder is recognized as the best nutrient source for acetoin fermentation using *Bacillus subtilis* (Gudiña et al. 2015; Tian et al. 2016). Given the abundant nutrients in okara, such as amino acids, vitamins, polypeptide, and minerals, acetoin fermentation using okara as a carbon source may reduce the required corn dry powder concentration. Figure 6 shows the effect of different concentrations of corn dry powder (0, 2.5, 7.5, 15, and 30 g/L) on acetoin fermentation. The engineered strain BS03 produced approximately 11.79 g/L acetoin without the addition of corn dry powder. The main reason for this was that cellulase and pectinase increased the nitrogen content in the medium during the enzymatic hydrolysis of okara (Fig. 2). In the fermentation of sugar mixtures simulating okara hydrolysate sugar composition (29 g/L total sugar) and supplemented with (2.5, 7.5, 15 g/L) corn dry powder and (2.5, 7.5, 15 g/L) yeast extract, respectively, the highest acetoin production and yield were obtained with 7.5 g/L of corn dry powder, with values comparable with those attained with okara hydrolysate (29 g/L reducing sugar) without supplementation of corn dry powder (Fig. 6b).

### Batch and fed-batch fermentations

**Fig. 7:**
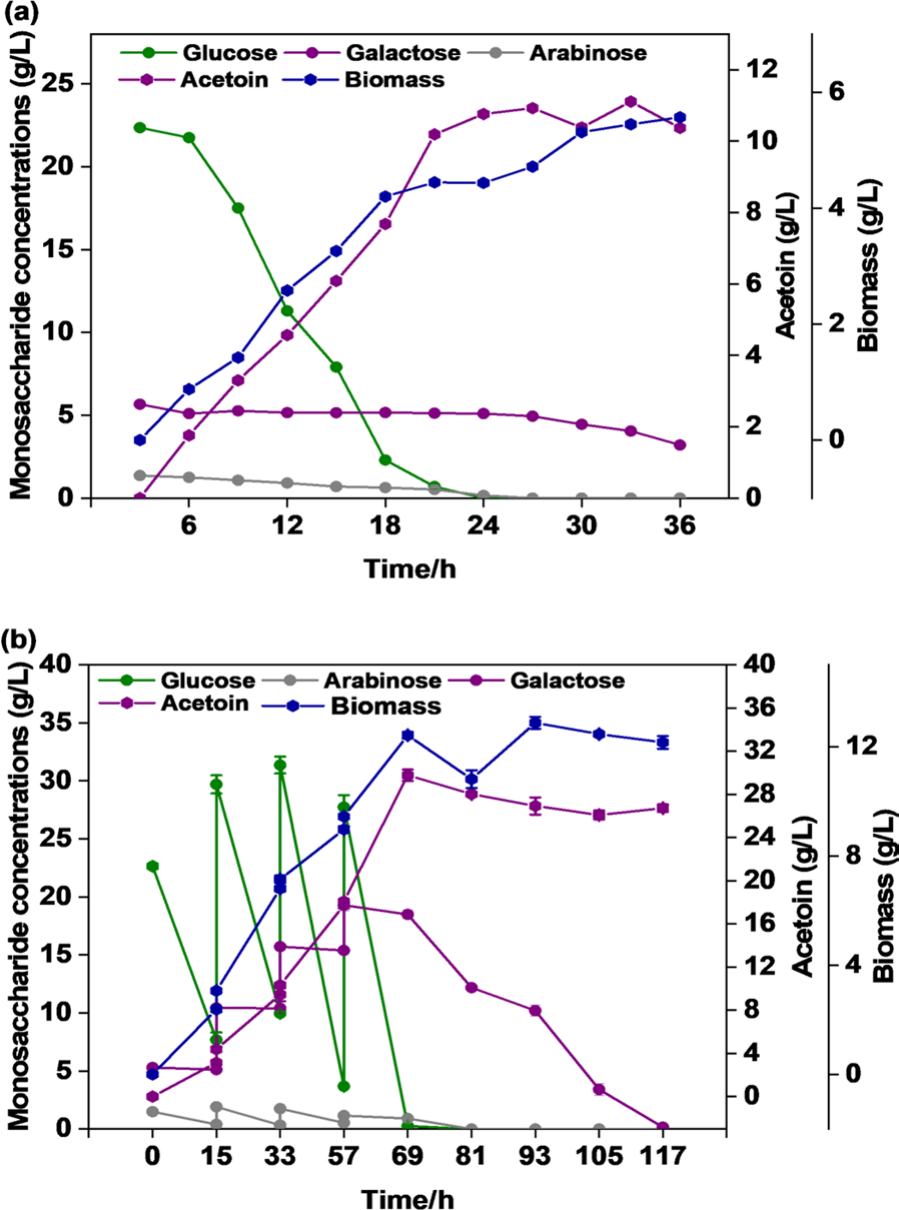
Batch fermentation **a** and fed-batch fermentation of recombinant strains in okara enzymatic hydrolysate medium to produce acetoin (**b**)

A pilot test of acetoin production was performed in a 7.5-L bioreactor using okara hydrolysates. The initial concentrations of glucose, galactose, and arabinose were 22.35, 5.31, and 1.36 g/L, respectively. As shown in Fig. 7a, the engineered strain BS03 showed sequential utilization of glucose–galactose. At 33 h, the engineered strain showed the highest fermentation yield of acetoin (11.11 g/L), which could be converted to TTMP (5.33 g/L). At this time, glucose and arabinose were completely consumed, and galactose was being consumed at 0.12 g/L/h. Cheng et al. (2011) showed that when glucose, arabinose, and galactose coexisted in the medium, the order of the mixed sugar utilized by *Bacillus subtilis* was glucose, arabinose, and galactose, which was also confirmed by our experimental results. The fermentation was completed at 36 h, when the galactose consumption was approximately 2.105 g/L and the acetoin yield decreased by 6.67%. To obtain a higher acetoin concentration, the BS03 strain was used for fed-batch fermentation, and approximately 29.7 g/L acetoin was obtained from 90.56 g/L glucose within 69 h, which was transformed into 13.37 g/L TTMP. At this point, 18.48 g/L of galactose and 0.905 g/L of arabinose remained. After extending the fermentation period to 117 h, galactose and arabinose were completely consumed, and the acetoin yield decreased by 10.3%.

## Discussion

The use of the renewable biomass okara as a carbon and nitrogen source for acetoin synthesis is crucial to the realization of commercial acetoin production. Dilute acid pre-treatment promoted in dissolution of cellulose and hydrolysis of hemicellulose, which led to increased sugar recovery in the liquid fraction and more available cellulose for enzymatic hydrolysis (Dionísio et al. 2021). Therefore, after dilute acid pretreatment and enzymatic hydrolysis, the concentration of arabinose and galactose in the hydrolyzed solution was significantly higher than in the untreated enzymatic hydrolysis solution (Table 2, Fig. 2). However, direct enzymatic digestion allows higher glucose concentrations without worrying about the production of inhibitors. In contrast to bagasse and corn cob, okara can be directly enzymatically digested to monosaccharides (Rastogi and Shrivastava 2022). In the former, it is necessary to remove lignin from the raw material or destroy its structure before cellulase can act effectively (Cai et al. 2016). Our results suggest that cellulase alone can hydrolyze cellulose from okara (Fig. 2a). In addition to cellulase, β-glucosidase, xylanase, and pectinase were also screened in the enzymatic hydrolysis reaction of okara. It is well known that cellobiose inhibits the action of exo-glucanase. If the cellulase complex does not have sufficient β-glucosidase activity, external supplementation is necessary to increase the enzyme efficiency (Pallapolu et al. 2011). To enhance the hydrolysis effect of commercial enzymes provided by Novozymes (Coppehagen, Denmark) on lignocellulose, hemicellulase is added to β-glucosidase, which led to an increase of galactose and arabinose concentrations in the hydrolysate. According to Kasai et al. (2004), the secondary cell wall of okara is a pectin-like substance that is resistant to hemicellulases, proteases, and even pectinases, which, explains why xylanase does not hydrolyze hemicellulose in okara. It has been shown that the okara primary cell wall can be easily digested by cellulase. However, only pectinase is capable of digesting the secondary cell wall, thereby releasing sugars and proteins (Kasai et al. 2003). Other components, such as lignin that cannot be hydrolyzed, remain in the enzymatic hydrolysate residue of okara. Figure 2 shows that pectinase can effectively hydrolyze hemicellulose and release free amino acids in okara. Finally, a hydrolsate rich in monosaccharides and amino acids was obtained by the combined hydrolysis of cellulase, β-glucosidase and pectinase.

Digestion of cellulose produces glucose and cellobiose, while digestion of hemicellulose produces mostly xylose and other sugars, such as galactose, mannose, arabinose, as secondary products (Kortei et al. 2014). D-galactose is abundant as a renewable biological resource in terrestrial plants, marine macroalgae, and dairy waste (Beniwal et al. 2021). Moreover, galactose content ranks second following glucose in okara enzymatic hydrolysate. To improve the galactose utilization efficiency in the acetoin-producing BS03 strain, the arabinose transporter protein (*araE*) and the galactose metabolism gene cluster *galKTE* were co-expressed in this study. Control strain BS01-pMA5 and BS02 exhibited no galactose degradation in mixed sugar media, while BS03 was affected by CCR and could only metabolize galactose after glucose depletion (Fig. 4c). CcpA, the global transcription factor of *Bacillus subtilis*, binds to the phosphorylated cofactor HPr to form a ternary complex that represses other carbon-derived catabolite related gene expression during mixed sugar fermentation (Sousa et al. 2019). It was suggested by Lu Lin et al. (2020) that CCR could be relieved by inhibiting the activities of two enzymes, CcpA (the global transcription factor of *Bacillus subtilis*) and HPrK. For example, Zhang et al. (2006) knocked down CcpA to alleviate CCR and improved riboflavin fermentation yields. Acetoin production from CcpA mutants was greatly reduced because CcpA is essential for AlsS expression (Sprehe et al. 2007). To reduce the CCR effect, we attempted to knock out the PTS system to improve the co-utilization efficiency of glucose and galactose, but the results showed that the CCR effect was not solely dependent on the PTS system. The results of the fermentation with the BS03 strain using different substrates (glucose, galactose, arabinose, xylose) to produce acetoin showed that the conversion efficiency of galactose to acetoin was low (Fig. 5b). Galactose usage requires four enzyme reactions to enter into the glycolytic pathway from glucose-6-phosphate. Consumption of d-glucose was faster than consumption of d-galactose. Approximately 72 h were required to ferment 6.01 g/L d-galactose, more than which is longer than is needed to ferment 10 g/L d-glucose (Fig. 5a, b). The slower metabolism could be one of the reasons low acetoin concentrations was produced from the fermentation of d-galactose. Liu et al. (2011) isolated the *Bacillus licheniformis* strain MEL09 from soil for use as an acetoin producer and found that fermentation with 60 g/L galactose resulted in only 0.06 g/L acetoin. The sugar concentration in okara enzymatic hydrolyzate and corn dry powder was optimized to increase acetoin yield. Even without nitrogen supplementation, the acetoin fermentation yield was only 3.4% lower than the highest yield. Considering the cost of the fermentation medium, corn dry powder was not added. Peptides, amino acids, and proteins released during the enzymatic digestion of okara provided sufficient nitrogen for acetoin production. Batch and fed-batch fermentation of okara enzymatic hydrolyzate with an initial concentration of 29 g/L sugar was performed in a 7.5 L bioreactor. The recombinant strain did not suffer from significant growth inhibition, which can be attributed to using milder enzymatic hydrolysis process to produce the okara hydrolysate. When a mixture of glucose, galactose, and arabinose was present, glucose was consumed by the BS03 strain first. Additionally, the expression of the genes involved in galactose metabolism is repressed in the presence of arabinose, when the glucose had been entirely consumed, arabinose was utilized for cell growth and acetoin production, and galactose was used as the carbon source. The low consumption rate of galactose after glucose depletion may directly affect the synthesis of acetoin. A possible explanation for this problem might be the limited expression levels of galactose metabolic genes in the engineered strain during acetoin fermentation. On the other hand, the arabinose transporter of *Bacillus subtilis* has different affinities for arabinose, xylose, and galactose, and this may cause the galactose uptake rate to be lower than xylose and arabinose (Geng et al. 2022).

According to our research, the food processing waste okara is a good substrate for producing acetoin and TTMP. Compared to agricultural waste, such as straw and corn cobs, food processing by-products like okara are readily available throughout the year, while agricultural waste requires seasonal considerations (Mardawati et al. 2018). Despite the low acetoin concentration synthesized by *Bacillus subtilis* from galactose, this research provides a reference for *Bacillus subtilis* in converting galactose to other bioproducts.

## Supplementary Information

**Additional file 1: Table S1.** Primers used in this study. **Table S2.** Amino acid composition of okara hydrolysate. **Fig. S1. a**–**c** Mutations of *yyzE* (**a**), *ypqe* (**b**), and *ptsG* (**c**) in BS01. **Fig. S2. a**, **b** Mutation of *bdhA* (**a**) and *acoA* (**b**).

## Data Availability

The obtained data will be available from the corresponding author upon reasonable request.

## References

[CR1] Beniwal A, Saini P, De S, Vij S (2021) Harnessing the nutritional potential of concentrated whey for enhanced galactose flux in fermentative yeast. LWT 141:110840. 10.1016/j.lwt.2020.110840 10.1016/j.lwt.2020.110840

[CR2] Cai D, Dong Z, Wang Y, Chen C, Li P, Qin P, Wang Z, Tan T (2016) Co-generation of microbial lipid and bio-butanol from corn cob bagasse in an environmentally friendly biorefinery process. Biores Technol 216:345–351. 10.1016/j.biortech.2016.05.073 10.1016/j.biortech.2016.05.073 27259190

[CR3] Chen T, Liu WX, Fu J, Zhang B, Tang YJ (2013) Engineering *Bacillus subtilis* for acetoin production from glucose and xylose mixtures. J Biotechnol 168(4):499–505. 10.1016/j.jbiotec.2013.09.020 24120578 10.1016/j.jbiotec.2013.09.020

[CR4] Cheng H, Wang B, Lv J, Jiang M, Lin S, Deng Z (2011) Xylitol production from xylose mother liquor: a novel strategy that combines the use of recombinant *Bacillus subtilis* and *Candida **maltosa*. Microb Cell Fact 10(1):5. 10.1186/1475-2859-10-5 21299871 10.1186/1475-2859-10-5 PMC3046924

[CR5] Choi IS, Kim YG, Jung JK, Bae H-J (2015) Soybean waste (okara) as a valorization biomass for the bioethanol production. Energy 93:1742–1747. 10.1016/j.energy.2015.09.093 10.1016/j.energy.2015.09.093

[CR6] Cui Z, Wang Z, Zheng M, Chen T (2021) Advances in biological production of acetoin: a comprehensive overview. Crit Rev Biotechnol 42(8):1135–1156. 10.1080/07388551.2021.1995319 34806505 10.1080/07388551.2021.1995319

[CR7] Ding C, Wang X, Li M (2019) Evaluation of six white-rot fungal pretreatments on corn stover for the production of cellulolytic and ligninolytic enzymes, reducing sugars, and ethanol. Appl Microbiol Biotechnol 103(14):5641–5652. 10.1007/s00253-019-09884-y 31115636 10.1007/s00253-019-09884-y

[CR8] Dionísio SR, Santoro DCJ, Bonan CIDG, Soares LB, Biazi LE, Rabelo SC, Ienczak JL (2021) Second-generation ethanol process for integral use of hemicellulosic and cellulosic hydrolysates from diluted sulfuric acid pretreatment of sugarcane bagasse. Fuel 304:121290. 10.1016/j.fuel.2021.121290 10.1016/j.fuel.2021.121290

[CR9] Geng B, Jia X, Peng X, Han Y (2022) Biosynthesis of value-added bioproducts from hemicellulose of biomass through microbial metabolic engineering. Metab Eng Commun 15:e00211. 10.1016/j.mec.2022.e00211 36311477 10.1016/j.mec.2022.e00211 PMC9597109

[CR10] Gudiña EJ, Fernandes EC, Rodrigues AI, Teixeira JA, Rodrigues LR (2015) Biosurfactant production by *Bacillus subtilis* using corn steep liquor as culture medium. Front Microbiol. 10.3389/fmicb.2015.00059 25705209 10.3389/fmicb.2015.00059 PMC4319461

[CR11] Hao N, Mu J, Hu N, Xu S, Shen P, Yan M, Li Y, Xu L (2016) Implication of ornithine acetyltransferase activity on l-ornithine production in *Corynebacterium **glutamicum*. Biotechnol Appl Biochem 63(1):15–21. 10.1002/bab.1353 25630515 10.1002/bab.1353

[CR12] Hu F, Liu Y, Lin J, Wang W, Li S (2020) Efficient production of surfactin from xylose-rich corncob hydrolysate using genetically modified *Bacillus subtilis* 168. Appl Microbiol Biotechnol 104(9):4017–4026. 10.1007/s00253-020-10528-9 32172322 10.1007/s00253-020-10528-9

[CR13] Jia X, Peng X, Liu Y, Han Y (2017) Conversion of cellulose and hemicellulose of biomass simultaneously to acetoin by thermophilic simultaneous saccharification and fermentation. Biotechnol Biofuels 10(1):232. 10.1186/s13068-017-0924-8 29046719 10.1186/s13068-017-0924-8 PMC5635544

[CR14] Jia X, Kelly RM, Han Y (2018) Simultaneous biosynthesis of (R)-acetoin and ethylene glycol from D-xylose through in vitro metabolic engineering. Metab Eng Commun 7:e00074. 10.1016/j.mec.2018.e00074 30197863 10.1016/j.mec.2018.e00074 PMC6127078

[CR15] Kasai N, Imashiro Y, Morita N (2003) Extraction of soybean oil from single cells. J Agric Food Chem 51(21):6217–6222. 10.1021/jf034130d 14518947 10.1021/jf034130d

[CR16] Kasai N, Murata A, Inui H, Sakamoto T, Kahn RI (2004) Enzymatic high digestion of soybean milk residue (Okara). J Agric Food Chem 52(18):5709–5716. 10.1021/jf035067v 15373413 10.1021/jf035067v

[CR17] Kortei NK, Dzogbefia VP, Obodai M (2014) Assessing the Effect of Composting Cassava Peel Based Substrates on the Yield, Nutritional Quality, and Physical Characteristics of Pleurotus ostreatus (Jacq. ex. Fr) Kummer. Biotechnol Res Int 2014:571520. 10.1155/2014/571520 25580299 10.1155/2014/571520 PMC4281436

[CR18] Krispin O, Allmansberger R (1998) The *Bacillus subtilis **galE* Gene Is Essential in the Presence of Glucose and Galactose. J Bacteriol 180(8):2265–2270. 10.1128/JB.180.8.2265-2270.1998 9555917 10.1128/JB.180.8.2265-2270.1998 PMC107161

[CR19] Li T, Zhan C, Guo G, Liu Z, Hao N, Ouyang P (2021) Tofu processing wastewater as a low-cost substrate for high activity nattokinase production using *Bacillus subtilis*. BMC Biotechnol 21(1):57. 10.1186/s12896-021-00719-1 34620130 10.1186/s12896-021-00719-1 PMC8499530

[CR20] Liu Y, Zhang S, Yong Y-C, Ji Z, Ma X, Xu Z, Chen S (2011) Efficient production of acetoin by the newly isolated *Bacillus licheniformis* strain MEL09. Process Biochem 46(1):390–394. 10.1016/j.procbio.2010.07.024 10.1016/j.procbio.2010.07.024

[CR21] Lu Lin XL, Liu Y, Guocheng Du, Chen J, Liu L (2020) Advances in design, construction and applications of *Bacillus subtilis* chassis cells. Synth Biol J 1(2):247–265

[CR22] Mardawati E, Andoyo R, Syukra KA, Kresnowati M, Bindar Y (2018) Production of xylitol from corn cob hydrolysate through acid and enzymatic hydrolysis by yeast. IOP Conf Series Earth Environ Sci 141:012019. 10.1088/1755-1315/141/1/012019 10.1088/1755-1315/141/1/012019

[CR23] Martín C, Galbe M, Wahlbom CF, Hahn-Hägerdal B, Jönsson LJ (2002) Ethanol production from enzymatic hydrolysates of sugarcane bagasse using recombinant xylose-utilising Saccharomyces cerevisiae. Enzyme Microbial Technol 31(3):274-282. 10.1016/S0141-0229(02)00112-6

[CR24] Moutta RO, Chandel AK, Rodrigues RCLB, Silva MB, Rocha GJM, Silva SS (2012) Statistical optimization of sugarcane leaves hydrolysis into simple sugars by dilute sulfuric acid catalyzed process. Sugar Tech 14(1):53–60. 10.1007/s12355-011-0116-y 10.1007/s12355-011-0116-y

[CR25] Pallapolu VR, Lee YY, Garlock RJ, Balan V, Dale BE, Kim Y, Mosier NS, Ladisch MR, Falls M, Holtzapple MT, Sierra-Ramirez R, Shi J, Ebrik MA, Redmond T, Yang B, Wyman CE, Donohoe BS, Vinzant TB, Elander RT, Hames B, Thomas S, Warner RE (2011) Effects of enzyme loading and β-glucosidase supplementation on enzymatic hydrolysis of switchgrass processed by leading pretreatment technologies. Biores Technol 102(24):11115–11120. 10.1016/j.biortech.2011.03.085 10.1016/j.biortech.2011.03.085 21507624

[CR26] Peng K, Guo D, Lou Q, Lu X, Cheng J, Qiao J, Lu L, Cai T, Liu Y, Jiang H (2020) Synthesis of ligustrazine from acetaldehyde by a combined biological-chemical approach. ACS Synth Biol 9(11):2902–2908. 10.1021/acssynbio.0c00113 33156612 10.1021/acssynbio.0c00113

[CR27] Rastogi M, Shrivastava S (2022) Assessment of indigenous fungal biocatalysts towards valorization of delignified physico-chemically pretreated corn cobs and sugarcane bagasse. Biofuels, Bioprod Biorefin. 10.1002/bbb.2435 10.1002/bbb.2435

[CR28] Redondo-Cuenca A, Villanueva-Suárez MJ, Mateos-Aparicio I (2008) Soybean seeds and its by-product okara as sources of dietary fibre, Measurement by AOAC and Englyst methods. Food Chem 108(3):1099–1105. 10.1016/j.foodchem.2007.11.061 26065777 10.1016/j.foodchem.2007.11.061

[CR29] Sousa J, Westhoff P, Methling K, Lalk M (2019) The absence of pyruvate kinase affects glucose-dependent carbon catabolite repression in *Bacillus subtilis*. Metabolites 9(10):216. 10.3390/metabo9100216 31590319 10.3390/metabo9100216 PMC6835821

[CR30] Souza BCd, Bossardi FF, Furlan GR, Folle AB, Reginatto C, Polidoro TA, Carra S, Silveira MMd, Malvessi E (2021) Validated high-performance liquid chromatographic (HPLC) method for the simultaneous quantification of 2,3-butanediol, glycerol, acetoin, ethanol, and phosphate in microbial cultivations. Anal Lett 54(15):2395–2410. 10.1080/00032719.2020.1869754 10.1080/00032719.2020.1869754

[CR31] Sprehe M, Seidel G, Diel M, Hillen W (2007) CcpA Mutants with Differential Activities in *Bacillus subtilis*. Microbial Physiology 12(1–2):96–105. 10.1159/000096464 10.1159/000096464 17183216

[CR32] Su M, Hu Y, Cui Y, Wang Y, Yu H, Liu J, Dai W, Piao C (2021) Production of β-glucosidase from okara fermentation using *Kluyveromyces**marxianus*. J Food Sci Technol 58(1):366–376. 10.1007/s13197-020-04550-y 33505081 10.1007/s13197-020-04550-y PMC7813905

[CR33] Syaftika N, Matsumura Y (2018) Comparative study of hydrothermal pretreatment for rice straw and its corresponding mixture of cellulose, xylan, and lignin. Biores Technol 255:1–6. 10.1016/j.biortech.2018.01.085 10.1016/j.biortech.2018.01.085 29414153

[CR34] Tian Y, Fan Y, Liu J, Zhao X, Chen W (2016) Effect of nitrogen, carbon sources and agitation speed on acetoin production of *Bacillus subtilis* SF4-3. Electron J Biotechnol 19:41–49. 10.1016/j.ejbt.2015.11.005 10.1016/j.ejbt.2015.11.005

[CR35] Vojcic L, Despotovic D, Martinez R, Maurer K-H, Schwaneberg U (2012) An efficient transformation method for *Bacillus subtilis* DB104. Appl Microbiol Biotechnol 94(2):487–493. 10.1007/s00253-012-3987-2 22395911 10.1007/s00253-012-3987-2

[CR36] Vong WC, Liu S-Q (2016) Biovalorisation of okara (soybean residue) for food and nutrition. Trends Food Sci Technol 52:139–147. 10.1016/j.tifs.2016.04.011 10.1016/j.tifs.2016.04.011

[CR37] Xiao Z, Lu JR (2014) Strategies for enhancing fermentative production of acetoin: A review. Biotechnol Adv 32(2):492–503. 10.1016/j.biotechadv.2014.01.002 24412764 10.1016/j.biotechadv.2014.01.002

[CR38] Xu Q, Wu J, Zou L, Ouyang J, Zheng Z (2022) Development of a process for the enhanced enzymatic digestibility of solid waste from tofu to yield fermentable biosugars. Biocatal Biotransform 40(1):64–74. 10.1080/10242422.2020.1865932 10.1080/10242422.2020.1865932

[CR39] Zhang F, Song H, Ban R (2006) Knockout of the HprK Gene in *Bacillus subtilis* CcpA Mutant and Its Influence on Riboflavin Fermentation. Chin J Biotechnol 22(4):534–538. 10.1016/S1872-2075(06)60042-3 10.1016/S1872-2075(06)60042-3 16894883

[CR40] Zhang B, Li XL, Fu J, Li N, Wang Z, Tang YJ, Chen T (2016) Production of acetoin through simultaneous utilization of glucose, xylose, and arabinose by engineered *Bacillus subtilis*. PLoS ONE 11(7):e0159298. 10.1371/journal.pone.0159298 27467131 10.1371/journal.pone.0159298 PMC4965033

[CR41] Zhou C, Shi L, Ye B, Feng H, Zhang J, Zhang R, Yan X (2017) *pheS**, an effective host-genotype-independent counter-selectable marker for marker-free chromosome deletion in *Bacillus **amyloliquefaciens*. Appl Microbiol Biotechnol 101(1):217–227. 10.1007/s00253-016-7906-9 27730334 10.1007/s00253-016-7906-9

