# Correction to: Production of acetoin and its derivative tetramethylpyrazine from okara hydrolysate with *Bacillus subtilis*

**DOI:** 10.1186/s13568-024-01757-6

**Published:** 2024-09-16

**Authors:** Tao Li, Ping Liu, Gege Guo, Zhaoxing Liu, Lei Zhong, Lianxia Guo, Cheng Chen, Ning Hao, Pingkai Ouyang

**Affiliations:** State Key Laboratory of Materials-Oriented Chemical Engineering, Jiangsu National Synergetic Innovation Center for Advanced Materials (SICAM), College of Biotechnology and Pharmaceutical Engineering, Nanjing Tech University, Nanjing, 211816 China

**Correction to: AMB Express (2023) 13:25**

10.1186/s13568-023-01532-z

Following publication of the original article (Li et al. 2023), the authors regret for the inadvertently use of duplicate images Fig. 3a1 and c1. The following figure shows the corrected Fig. 3 in which the image of (a1) is replaced with a new one. As a negative control image, this change does not affect any other results or the conclusions associated with this article.

**Fig. 3:**
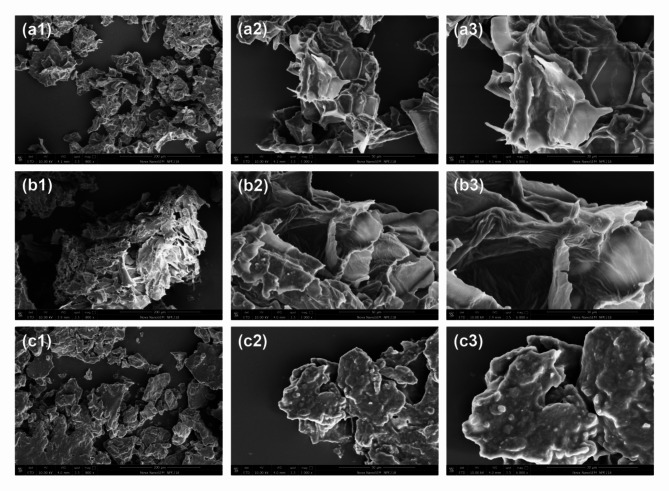
SEM images of okara samples, either untreated dry okara (**a1**–**a3**) or treated using cellulase (**b1**–**b3**) and cellulase + β-glucosidase + pectinase (**c1**–**c3**). The magnification and scale bars are provided in each micrograph
