# Supplementary material for: Production of acetoin and its derivative tetramethylpyrazine from okara hydrolysate with Bacillus subtilis
Source: AMB Express. 2023 Feb 28;13:25. doi: 10.1186/s13568-023-01532-z (PMC9975146; doi:10.1186/s13568-023-01532-z)
Supplement: Supplementary file 1 — Additional file 1: Table S1. Primers used in this study. Table S2. Amino acid composition of okara hydrolysate. Fig. S1. a–c Mutations of yyzE (a), ypqe (b), and ptsG (c) in BS01. Fig. S2. a, b Mutation of bdhA (a) and acoA (b). [file 13568_2023_1532_MOESM1_ESM.docx]

Additional file 1

**Table S1 Primers used in this study**

| Primer | Sequence |
| --- | --- |
| PC-F | ATTTTTAAAGTATGTATACAAATGA |
| PC-R | TTATAAAAGCCAGTCATTAGGCCTA |
| acoA-LF-F | cttatgtgaaacaaacctatcctaagaaagaaatcaaga |
| acoA-LF-R | ttttaaaaccttgatttgtgcgcctcctt |
| acoA-DR-F | ggaggcgcacaaatcaaggttttaaaaa |
| acoA-DR-R | TATACATACTTTAAAAATcctgatcaaagcacgtgc |
| acoA-PC-F | gcacgtgctttgatcaggATTTTTAAAGTATGT |
| acoA-PC-R | cagttaatgacaagccttTTATAAAAGCCAGTC |
| acoA-RF-F | ATGACTGGCTTTTATAAaaggcttgtcattaac |
| acoA-RF-R | atggcatctttttcttcaaggtgct |
| bdhA-LF-F | ctgtaatgccccgctactttacc |
| bdhA-LF-R | gtctgttttagacgggattaccactccta |
| bdhA-DR-F | aggagtggtaatcccgtctaaaacagacg |
| bdhA-DR-R | TACATACTTTAAAAATggaaaacttccgcgct |
| bdhA-PC-F | agcgcggaagttttccATTTTTAAAGTATGT |
| bdhA-PC-R | tcttgctgccttcatTTATAAAAGCCAGTC |
| bdhA-RF-F | GACTGGCTTTTATAAatgaaggcagcaaga |
| bdhA-RF-R | ctgacgatgacggtttcaccg |
| ptsG-LF-F | ctaaagaaccagaaaaattaatgttgttattgaaaaatgaatatccg |
| ptsG-LF-R | tgcagaatctcataaggaacaagataaccgataa |
| ptsG-DR-F | attatcggttatcttgttccttatgagattctgc |
| ptsG-DR-R | GTATACATACTTTAAAAATcgatcgcatgttttgtcg |
| ptsG-PC-F | cgacaaaacatgcgatcgATTTTTAAAGTATGT |
| ptsG-PC-R | cactcatggatacattcattTTATAAAAGCCAGTC |
| ptsG-RF-F | CTAATGACTGGCTTTTATAAaatgaatgtatccatgag |
| ptsG-RF-R | atcttaacattcaacagctgcatgacc |
| yyzE-LF-F | gatttcgttgtttaaatacgtcacgatgga |
| yyzE-LF-R | atgtttaattcctccgacaaccgtaatgac |
| yyzE-DR-F | gtcattacggttgtcggaggaattaaacat |
| yyzE-DR-R | GTATACATACTTTAAAAATgggtaaagcaagcct |
| yyzE-PC-F | cagaggcttgctttacccATTTTTAAAGTATGT |
| yyzE-PC-R | ttgtaggcgtaaccatTTATAAAAGCCAGTC |
| yyzE-RF-F | GACTGGCTTTTATAAatggttacgcctaca |
| yyzE-RF-R | gattgttcacatccatttggttgttaatctcg |
| ypqe-LF-F | ccgatccagcctatgcctatg |
| ypqe-LF-R | gcatacgccctgctgtattttctccctt |
| ypqe-DR-F | ggagaaaatacagcagggcgtatgcct |
| ypqe-DR-R | ATCATTTGTATACATACTTTAAAAATaaagcttttaatatcaatgccc |
| ypqe-PC-F | aaatgggcattgatattaaaagctttATTTTTAAAGTATGTATACAA |
| ypqe-PC-R | ccgaataattttttcagcaaTTATAAAAGCCAGTC |
| ypqe-RF-F | CTAATGACTGGCTTTTATAAttgctgaaaaaattattcgg |
| ypqe-RF-R | tgaaaagcggaatattggtgagtatatagttcat |
| araE-F | aaagtgaaatcagggggatccATGAAGAATACTCCAACTCAATTAGAACC |
| araE-R | atttcgacctctagaacgcgtTCATTTTATCCAAAGCTTTTCAATTT |
| F-araE | aaagtgaaatcagggggatccATGAAGAATACTCCAACTCAATTAGAACC |
| R-araE | gcaagggacaggtagtaTCATTTTATCCAAAGCTTTTCAATTT |
| F-HpaII | tgaTACTACCTGTCCCTTGCTGATTTTT |
| R-HpaII | gtaaatcATGTAAATCGCTCCTTTTTAGGTGG |
| F-galKTE | gagcgatttacatGATTTACATATGAGAGTTCTGGTTACCG |
| R-galKTE | atttcgacctctagaacgcgtTCAGCACTGTCCTGCTCCTTG |

**Table S2 Amino acid composition of okara hydrolysate**

| Amino acid | Concentration (g/L) |
| --- | --- |
| Aspartic acid (Asp) | 1.31 |
| Glutamate (Glu) | 0.86 |
| Serine (Ser) | 0.65 |
| Threonine (Thr) | 1.16 |
| Alanine (Ala) | 0.801 |
| Proline (Pro) | 0.57 |
| Tyrosine (Tyr) | 0.93 |
| Valine (Val) | 0.7 |
| Isoleucine (Ile) | 0.379 |
| Leucine (Leu) | 1.59 |
| Phenylalanine (Phe) | 1.04 |
| Lysine (Lys) | 2.22 |
| Total | 12.21 |

**Table S3 Sugar and total amino acid concentrations under different enzyme combinations**

| Enzyme loading | Glucose(g/L) | Arabinose(g/L) | Galactose(g/L) | Total amino acid(g/L) |
| --- | --- | --- | --- | --- |
| 20 FPU/ml Cellulase | 30.84±0.17 | <0.1 | < 0.1 | 3.49±0.06 |
| 20 FPU/mlCellulase+3 FBG/ml β-glucosidase | 33.12±0.09 | 0.74±0.02 | 4.26±0.08 | 4.26±0.08 |
| 20 FPU/mlCellulase+3 FBG/ml β-glucosidase+383 PNG/ml Pectinase | 32.78±0.23 | 1.43±0.06 | 7.74±0.11 | 12.21±0.23 |

**Fig. S1**


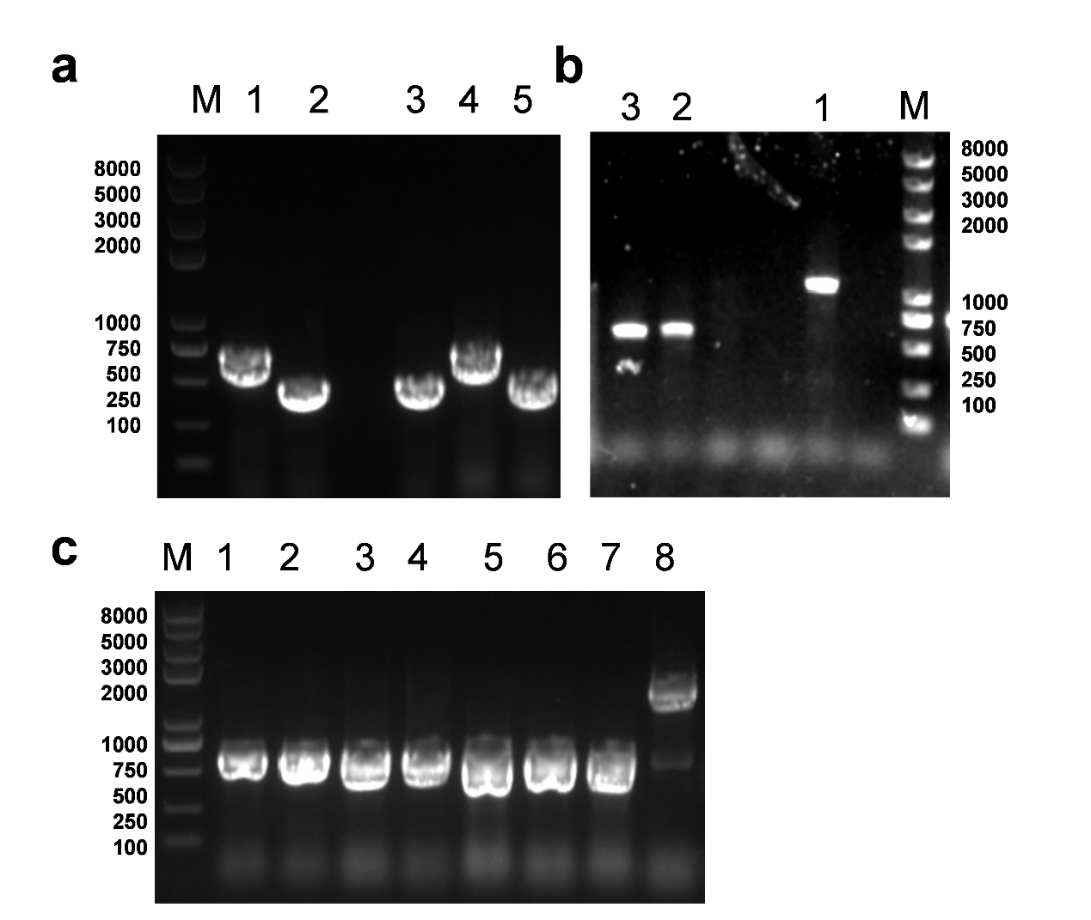


(a-c) Mutation of *yyzE*(a), *ypqe*(b) and *ptsG*(c) in BS01.

**Fig. S2**


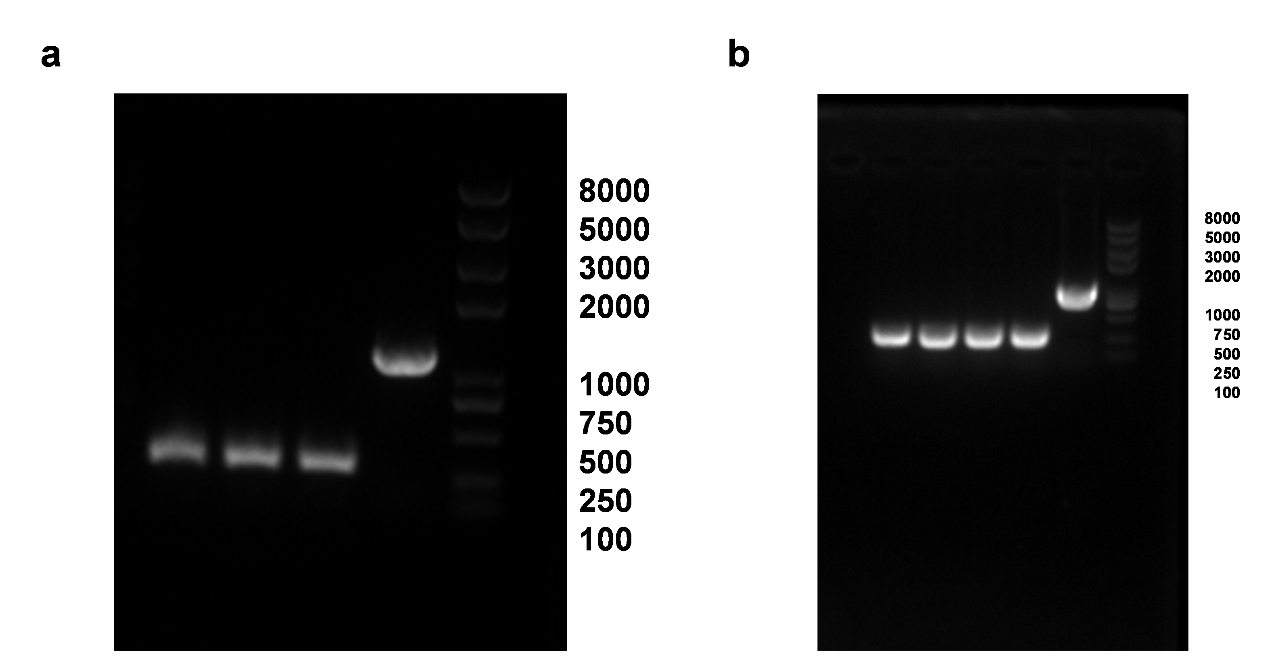


(a–b) Mutation of *bdhA* (a) and *acoA* (b)
